# Treatment of postsurgical trigeminal neuralgia with Fu’s subcutaneous needling therapy resulted in prompt complete relief: Two case reports

**DOI:** 10.1097/MD.0000000000033126

**Published:** 2023-03-03

**Authors:** Youling Gao, Jian Sun, Zhonghua Fu, Po-En Chiu, Li-Wei Chou

**Affiliations:** a School of Acupuncture, Moxibustion and Tuina, Nanjing University of Chinese Medicine, Nanjing, China; b Department of Acupuncture and Moxibustion, Yangzhou TCM Hospital Affiliated to Nanjing University of Chinese Medicine, Yangzhou, China; c Second Clinical Medical College, Guangzhou University of Chinese Medicine, Guangzhou, China; d Clinical Medical College of Acupuncture & Moxibustion and Rehabilitation, Guangzhou University of Chinese Medicine, Guangzhou, China; e Institute of Fu’s Subcutaneous Needling, Beijing University of Chinese Medicine, Beijing, China; f Department of Chinese Medicine, Chang Bing Show Chwan Memorial Hospital, Changhua, Taiwan; g Graduate Institute of Integrated Medicine, College of Chinese Medicine, China Medical University, Taichung, Taiwan; h Department of Physical Medicine and Rehabilitation, China Medical University Hospital, Taichung, Taiwan; i Department of Physical Therapy and Graduate Institute of Rehabilitation Science, China Medical University, Taichung, Taiwan; j Department of physical Medicine and Rehabilitation, Asia University Hospital, Asia University, Taichung, Taiwan.

**Keywords:** acupuncture, case report, Fu’s subcutaneous needling, pain, trigeminal neuralgia

## Abstract

Trigeminal neuralgia is a recurrent unilateral transient electroshock-like pain. Fu’s subcutaneous needling (FSN), used to treat the musculoskeletal problems, has not been reported in this field.

**Patient concerns:**

The pain extent of case 1 had no reduction after the previous microvascular decompression, the pain of case 2 relapsed 4 years after the microvascular decompression.

**Diagnoses:**

Postsurgical trigeminal neuralgia.

**Interventions:**

FSN therapy was applied on the muscles around the neck and face area, which the myofascial trigger points were palpated in these muscles. The FSN needle was inserted into the subcutaneous layer and the needle tip was pointed toward the myofascial trigger point.

**Outcomes:**

The following outcome measurements were observed before and after treatment, including numerical rating scale, Barrow Neurology Institute Pain Scale scores, Constant Face Pain Questionnaire scores, Brief Pain Inventory-Facial scores, Patient Global Impression of Change scores, and medication dosage. The follow-up surveys were made after 2 and 4 months respectively. The pain of Case 1 was significantly reduced after 7 times FSN treatments and the pain of Case 2 was even disappeared after 6 times FSN treatments.

**Lessons:**

This case report suggested that FSN can relieve postsurgical trigeminal neuralgia safely and effectively. Clinical randomized controlled studies are needed to be further conducted.

## 1. Introduction

Trigeminal neuralgia (TN) is a disturbing chronic pain condition on 1 side of the face. *The International Classification of Headache Disorders and International Classification of Orofacial Pain* defined TN as “a recurrent unilateral transient electroshock-like pain that has a sudden onset and termination, limited to the distribution of 1 or more divisions of the trigeminal nerve and triggered by harmless stimuli.”^[1]^ Touching the face, talking, eating, or drinking can all cause fierce pain. The nature and severity of the pain maybe related to delayed diagnosis, fear of pain, side effects of medications, and lack of psychological support.^[2]^ European studies based on epidemiology and genetics have found that the lifetime prevalence of TN is 0.16% to 0.3%, and the incidence rate per 100,000 people a year is 12.6 to 27.0. TN affects women (60%) more than men (40%).^[3]^ TN patients have increased symptoms such as anxiety, depression, insomnia, affecting basic psychological, physical, social needs and activities of patients, and TN is even called suicide disease.^[1,4]^ Microvascular decompression (MVD) is the first-line surgical therapy for medically refractory patients. Lars Bendtsen et al^[3]^ reported that MVD had a good effect, and 62% to 89% of patients exhibited no pain at follow-up (3–11 years later), but Giorgio Cruccu hold different opinions, he reported about 50% of TN patients also have persistent pain in the same area in addition to the characteristic paroxysmal episodes.^[5]^

Fu’s subcutaneous needling (FSN) therapy is a new type of acupuncture therapy developed in 1996. With disposable FSN needles, FSN mainly stimulates the subcutaneous layer adjacent to myofascial trigger points (MTrPs),^[6]^ which are hard, discrete, and palpable nodules in a taut band of skeletal muscle. FSN therapy is often performed by the reperfusion approach, the process of contracting a muscle for seconds, combined with the swaying movement.^[7]^ FSN therapy has a reliable effect in reducing pain, especially musculoskeletal problems.^[8–12]^ However, so far, there is no report of FSN for TN. In this case report, we presented 2 cases of TN patients who have persist pain after the surgery of MVD. The symptoms were surprising significant relieved or eliminated after several sessions of FSN treatments.

## 2. Case presentation

Two TN patients who received MVD surgery previously were treated by FSN therapy in Yangzhou TCM Hospital, East China, from October 2020 to April 2021. The authors obtained the written consents of the patients to describe their illness and willingness to publish their case reports. We did not use patient data that could allow their identification.

**Figure 1.**
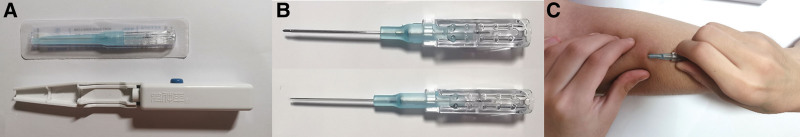
A. FSN needle and insertion device; B. The steel needle was pulled back 3 mm to select the protrusion of the cannula handle clockwise and to fix it in a slot; C. The FSN needle inserted into the subcutaneous layer. FSN = Fu’s subcutaneous needling.

Disposable FSN needles (Fig. 1A, from Nanjing FSN Medical Co., Ltd.) were used for FSN therapy.^[7]^ The patients lied in a supine position. We palpated the MTrPs with the relevant muscles around the neck and face area, and then marked them. After locating the MTrPs, chose the insertion points around the MTrP as the guidance of FSN practice.^[7]^ Each insertion point was disinfected with iodophor. The FSN needle (Fig. 1B) was inserted into the subcutaneous layer quickly through the skin. The needle tip was pointed toward the MTrP. The needle was pushed forwards until the whole cannula under the skin. The steel needle was pulled back 3 mm to fit the protrusion of the cannula handle clockwise and to fix it in a slot (Fig. 1B), so as to prevent the needle tip from damaging blood vessels or other tissues during the swaying movement (the needle movement from side to side horizontally, firmly, and rhythmically 200 times within 2 minutes) (Fig. 1C). During and immediately after the swaying movement, the results were tested by palpation or asking the patient opening the mouth, chewing or blowing to feel the effect. During the treatments, there was no broken needle, fainting, obvious pain, and nor other phenomena occurred.

Numerical rating scale (NRS)^[13]^ was used to evaluate the subjective pain intensity. The other 4 symptom scores included Barrow Neurology Institute Pain Scale score,^[13]^ Constant Face Pain Questionnaire score,^[14]^ Brief Pain Inventory-Facial score,^[15,16]^ and Patient Global Impression of Change score^[16,17]^ were used for the outcome measurements. The higher the score, the more severe the pain were. Besides, we also observed the medication dosage, the less dosage, and the better effect.

### 2.1. Clinical courses of case 1

**Figure 2.**
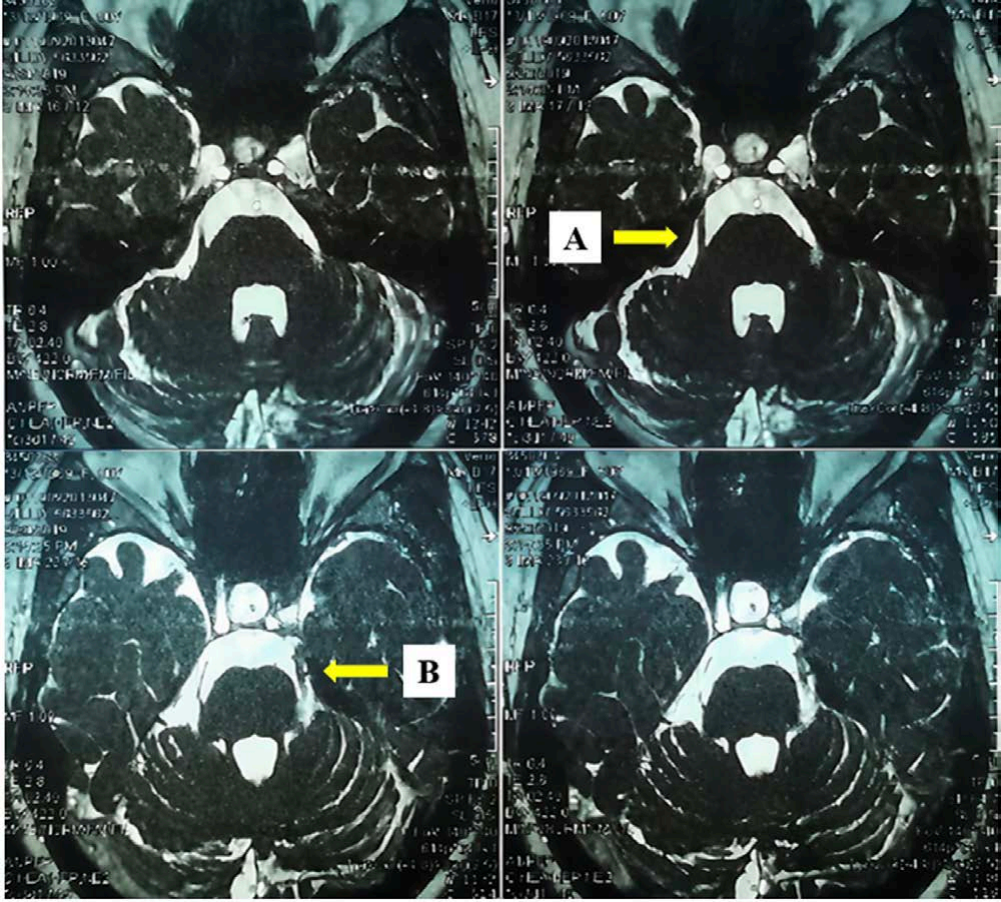
A. The right trigeminal nerve; B. The left trigeminal nerve degenerated.

On October 24, 2020, a 52-year-old woman visited our clinic. She received a hyperthyroidism operation 20 years ago, had anemia for 20 years. After receiving 2 MVD surgeries (2016-08-10, 2018-11-29) for her TN problems, her symptoms remained the same till then. The details about her medical history showed as Table 1. After these 2 operations, maxillofacial magnetic resonance tomographic angiography on September 20, 2019 showed degeneration of the left trigeminal nerve (Fig. 2).

**Table 1:** Clinical courses of case 1.

Time	Pain feature and factor	Treatments	Outcomes
13-Mar-2013	Left mandible pain root canal problems was suspected	Endodontic treatment	Pain referred to the middle of her upper and lower lips, could not squeeze her lips
In 2013	Oral problems were ruled out by the dentists trigeminal neuralgia was diagnosed	Painkillers were ineffective oral carbamazepine (600 mg/d)	A slight analgesic effect
Before operation	Dizzy, sleepy, unstable walking, and even felt severe pain and woke up as long as turning over and touching the left face during the sleeping time	Oral carbamazepine, Chinese medicine, traditional acupuncture, moxibustion, cupping	Ineffective treatment
10-Aug-2016	Upper and lower lips pain	MVD surgery	Pain was not relieved and referred to the left cheek
29-Nov-2018	Left cheek area (including buccal muscle) pain	MVD surgery, again	Worsened, pain often radiated to the left temporal region
24-Oct-2020	Mouth open < 1-finger wide (Fig. 2A) pain aggravated when opening mouth wider or speaking, chewing, or wind blowed and silk scarves touched	1st FSN treated the TMs of the left upper limb, neck, and shoulder	Outcomes measurement were noted in Table 2.
26-Oct-2020	Pain slightly reduced, could eat a small amount of food	Palpated the exact same TMs 2nd FSN treatment	Outcomes measurement were noted in Table 2.
28-Oct-2020	Frequency reduced; pain point fixed	3rd FSN treatment	During the third treatment, could open mouth 4 fingers wide (Fig. 2B)
30-Oct-2020	Frequency and degree reduced, sleep well last night	4th FSN treatment	Overall condition significantly relieved by 50%
3-Nov-2020	Occasionally pain, knead left eye, and pain happened from the dimple to the temporal area	5th FSN treatment	Overall condition significantly relieved by 80%–90%
6-Nov-2020	Several radiation-like pain, degree reduced	6th FSN treatment	Overall condition significantly relieved by 70%–80%
10-Nov-2020	Trigger point no radiation-like pain any more, only a little sour pain	7th FSN treatment	Overall condition significantly relieved by 90%

FSN = Fu’s subcutaneous needling, MVD surgery = microvascular decompression surgery, TMs = tightened muscles.

The NRS was 8 during rest time, and reached to 10 while opening mouth, chewing, talking, and blowing at the first visit. The local facial muscles were bruised due to longtime massage by herself. The patient felt soreness during palpation of the sternocleidomastoid muscle on the left side of the neck and the left buccal muscle. A lot of active MTrPs were found in the left biceps, trapezius, scalene, sternocleidomastoid, and buccal muscles while physical examination of her neck and upper back.

**Figure 3.**
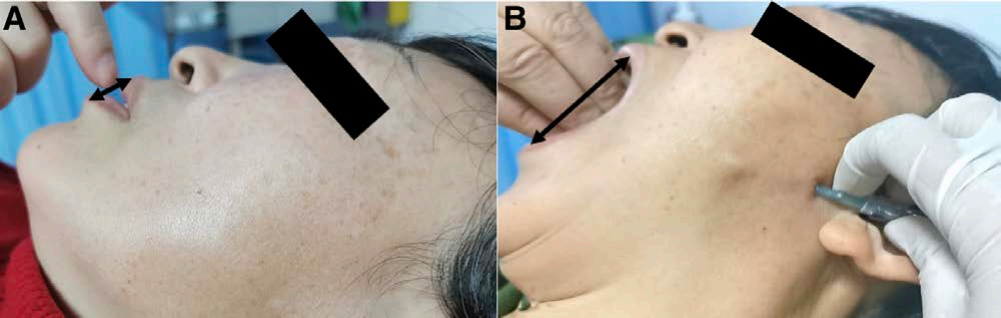
A. Mouth can open < 1 finger wide; B. Mouth can open 4 fingers immediately after 3 treatments of FSN. FSN = Fu’s subcutaneous needling

Seven sessions of FSN therapies were performed within 2 months. After treatments, her condition was significantly improved, and pain only occurred while in cold weather or in the fatigue state (Fig. 3). The evaluation scale for 7 treatments is listed in Table 2.

**Table 2:** Evaluation outcomes for 7 FSN treatments of case 1.

Date for FSN	NRS	BNI-PS	CFPQ	BPIF	PGIC	Dosage (Carbamazepine)
	(0–10)	(I–V)	(0–10)	(0–180)	(1–7)	
24-Oct-2020	10	V	10	155	6	0
26-Oct-2020	9	-	9	-	3	0
28-Oct-2020	7	-	7	-	2	0
30-Oct-2020	5	-	5	-	2	0
3-Nov-2020	3	-	2	-	1	0
6-Nov-2020	2	II	2	-	1	0
10-Nov-2020	1	I	0	0	1	0

NRS is 0 to 10 (10 is most pain); PGIC is 1-7 (7 is very much worse); Dosage is the use of drugs (Carbamazepine).

BNI-PS = Barrow Neurology Institute Pain Scale score, BPIF = Brief pain inventory-facial, CFPQ = Constant Face Pain Questionnaire, FSN = Fu’s subcutaneous needling, NRS = numerical rating scale, PGIC = patient global impression of change.

### 2.2. Clinical courses of case 2

**Figure 4.**
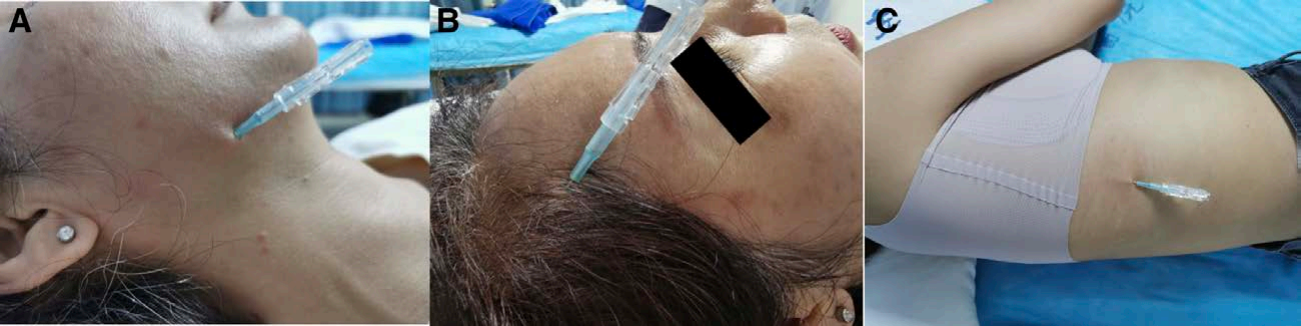
A. B. C. FSN needle points of masseter, galea aponeurotica and serratus anterior muscles. FSN = Fu’s subcutaneous needling.

A 46-year-old female with a history of Hashimoto thyroiditis under medicine for maintaining normal thyroid function and mild anemia under the medication of iron supplements orally was treated in our clinic. The details medical history is listed in Table 3. NRS was 7 at rest and increased to 10 when waking up and sitting up at the first visit. After 6 sessions of FSN therapies within 2 months, the patient recovered clinically (Fig. 4). The evaluation scales for 6 treatments are listed in Table 4.

**Table 3:** Clinical courses of case 2.

Time	Pain feature and factor	Treatments	Outcomes
16-Nov-2016	Tooth pain, needle-like pain	Remove the wisdom tooth	No improvement
16-Dec-2016	Paroxysmal needle-like pain on the right face trigeminal neuralgia was diagnosed	Several conservative treatments	Ineffective
Mar 9, 2017	Pain worsens	MVD surgery	Pains disappeared
20-Jan-2020	Relapsed for the 1st time	Gabapentin orally per d	The effect not obvious
			The symptoms relieved in 10 d
20-Jun-2020	Emotionally stressful events triggered the 2nd relapse	Oral oxcarbazepine (300 mg) up to 900 mg per d	Symptoms were relieved after more than 70 d
From Jan 1–Mar 3, 2021	Anxious and worried, 3rd relapse, particularly Severe for a period of time	Oral oxcarbazepine	Relieved
Mar 17, 2021	Started with a slight electric shock	Oral oxcarbazepine	Ineffective
26-Mar-2021	Persistent pain of flesh-picking, too severe to fall asleep, much worsened while lying down, and any physical activity or action would cause pain	Oral oxcarbazepine	Ineffective
29-Mar-2021	The pain interval was 1 min, could lie flat the night, but symptoms worsened when woke up and sit up, could eat and talk with no extra effort in the past 2 d. The pain point was not fixed, including the area of the right buccinator, temporalis, orbicularis oris muscle, and nasal muscles	1st FSN treated the TMs of the head and neck splenius, galea aponeurotica, and serratus anterior muscles. (Fig. 4)	The pain was immediately relieved after the first FSN treatment. After resting for half an h, felt the pain again, which stopped from time to time, could only speak when the pain was gone
30-Mar-2021	No pain after 8:00 pm after the first visit, took oxcarbazepine 450 mg again to prevent recurrence. Didn’t feel pain when turning over and sleeping, had pain when going to the toilet at night	2nd FSN treatment	The overall condition was significantly relieved
1-Apr-2021	No pain after waking up, felt a transient pain about 1min and the same degree as before, pain when washing face, brushing teeth, and eating. Pain point was from the upper midpoint of the orbicularis oris m to the right nasal m, levator lip nasalis m, and occasionally diverged to the right temporal m, took oxcarbazepine twice a d for a total of 300 mg	3rd FSN treatment	The medication was stopped
2-Apr-2021	Able to sleep at night, pain frequency was 70% lower overall	4th FSN treatment	Pain disappeared after the treatment, and the patient had facial pain twice during sleep
5-Apr-2021	Pain frequency became lower by 80%, felt pain twice in the morning, pain degree was almost the same, and the duration of pain became shorter	5th FSN treatment	No pain
6-Apr-2021	No pain	6th FSN treatment	No pain

FSN = Fu’s subcutaneous needling, m = muscle, MVD surgery = Microvascular decompression surgery, TMs = tightened muscle.

**Table 4:** Evaluation outcomes for 6 FSN treatments of case 2.

Date for FSN	NRS	BNI-PS	CFPQ	BPIF	PGIC	Dosage (Ocacepine)
	(0–10)	(I–V)	(0–10)	(0–180)	(1–7)	
29-Mar-2021	10	V	10	166	6	450
30-Mar-2021	7	III	7	-	3	300
1-Apr-2021	6	II	6	-	2	0
2-Apr-2021	3	II	3	-	2	0
5-Apr-2021	2	I	2	-	1	0
6-Apr-2021	0	I	0	0	1	0

NRS is 0 to 10 (10 is most pain); PGIC is 1-7 (7 is very much worse); Dosage is the use of drugs (Ocacepine).

BNI-PS = Barrow Neurology Institute Pain Scale score, BPIF = brief pain inventory-facial, CFPQ = Constant Face Pain Questionnaire, FSN = Fu’s subcutaneous needling, NRS = numerical rating scale, PGIC = patient global impression of change.

## 3. Discussion

**Figure 5.**
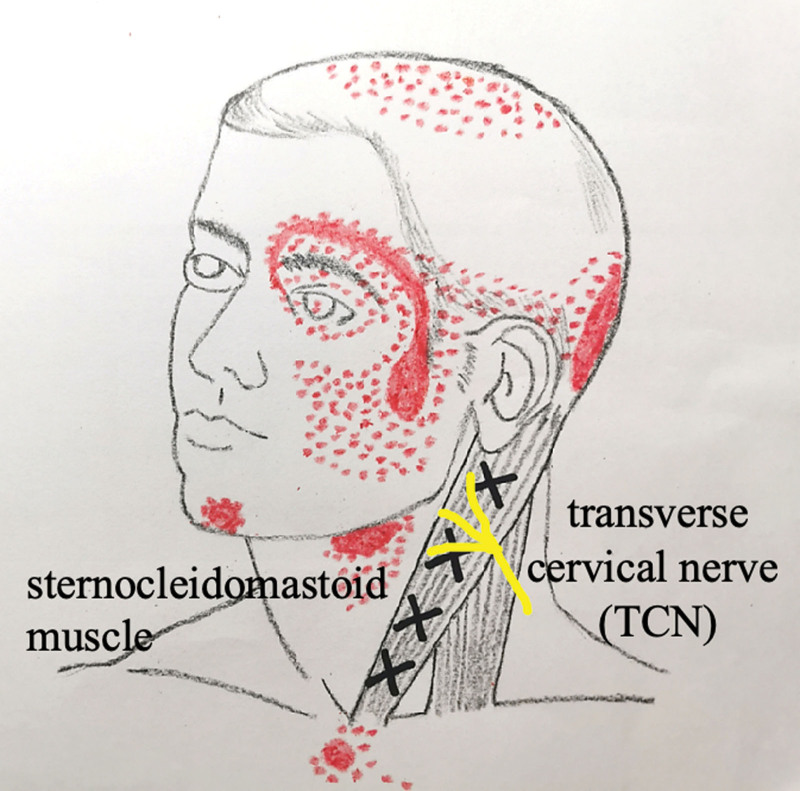
Referral pattern of the trigger point in the sternocleidomastoid muscle (red spots). Transverse cervical nerve (TCN) is in the yellow line.

TN is currently 1 of the neuropathic diseases that are difficult to treat. The pain is very severe that patients lose their confidence in life. Although many treatment methods are available (such as nervous system analgesics, local anesthetics, hormone therapy, and surgical treatment), and the effect is not satisfactory. Some can only temporarily relieve the pain, and others have many sequelae. Although the conditions of many patients initially improved during first-line treatment, most treatments tend to lose efficacy over time, so new treatment options are necessary.^[18]^ Previous studies have reported the effectiveness of FSN in the treatment of neck pain,^[19]^ low back pain,^[20–22]^ Lateral epicondylalgia^[12]^ and other painful diseases.^[23]^ The targets of treatment in the previous mentioned problems are MTrPs. In this article, the 2 cases are classified as typical TN according to the diagnostic classification. Myofascial trigger point (MTrP) is a major cause of muscle pain, characterized with a hyperirritable spot due to accumulation of sensitized nociceptors in skeletal muscle fibers.^[6]^ As shown in Figure 5, the referral pain pattern of the trigger point of the sternocleidomastoid muscle were reported to linked to the pain of head and face.^[24]^ The transverse cervical nerve, the skin below the chin area, that is, the transverse cervical nerve drawn in yellow on the front of the neck in Figure 5, can usually be referred to as atypical TN.^[24]^ The distribution of referred pain pattern and trigeminal nerve was almost similar, so our participants had treated the TN with FSN, and the results were unexpectedly good.

For the mechanism research of FSN therapy, Fu ZH et al^[21]^ have evaluated the end-plate noise recorded using the skeletal muscle myofascial trigger spot (MTrS) in rabbits to determine the electrophysiological effect of FSN. The evaluation of rabbit skeletal muscle MTrS by recording end-plate noise, which is equivalent to MTrP in human muscle, confirmed the effects and possible pathways of FSN on electrophysiological phenomena. After the ipsilateral distal FSN treatment, MTrS irritation seems to be suppressed, the proximal FSN treatment has a better effect than the distal treatment. Based on this study, and the combined proximal and distal treatment were performed in the 2 cases. The cases in this paper show that FSN also has a good effect on head and neck MTrPs, but there is no comparison between the proximal and distal treatment efficacy, which will be observed in the future. Therefore, in addition to examining the patient facial muscles, the head, neck, shoulders, upper limbs, and thoracodorsal muscles were examined for MTrPs. Case 2 is more complicated than Case 1. In Case 2, the MTrPs were detected in the head and neck splenius, galea aponeurotica, and serratus anterior muscles.

Antonio et al^[25]^ found that fascia tissue is related to the etiology of capsular neuropathy, emphasizing the importance of general connective tissues (except ligaments) in peripheral nerve compression. Although the pathogenesis of TN is still not fully understood, neurovascular conflict is still the most accepted theory at present.^[4]^ Many factors can make arteries narrow, such as arteritis, and arterial embolism, but the muscle with MTrPs is the most common factor that changes the diameter of arteries.^[7]^ The treatment mechanism of FSN is speculated to be similar to that of the above methods. FSN treatment aims to restore and enhance muscle function by eliminating MTrPs, alleviating muscle tightness and cramps, and reducing pain.^[6,7]^ These 2 cases of TN in this paper had left and right facial pain, and local tissue nutrition or energy supply was not available. When the action lasted for a period of time, the local blood supply was slightly relieved. When the muscles of the neck and facial on the ipsilateral side were too tight (i.e., the muscle with MTrPs squeezed the blood vessels and nerves passing through them), they affected the blood supply in the head and face.

The advantage of FSN therapy is to propose a targeted treatment plan on the basis of the individual differences of each patient. According to the anatomy and treatment experience, the MTrPs can be found on the ipsilateral head, face, neck, shoulders, and chest (e.g., galea aponeurotica, masseter, sternocleidomastoid, splenius capitis/cervicis, trapezius, and serratus anterior muscles). FSN therapy can greatly improve local microcirculation through swaying movement and reperfusion approaches, enhancing the nutrient supply and blood flow of the distribution of the trigeminal nerve and the surrounding soft tissues, improving or eliminating local inflammation and edema, and realizing a significant relief in symptoms.^[7]^

The cases in this report had limitations as follows. First, there was only a few treatment cases were observed. Secondary, there was no more accurate laboratory examination, like MRI to be conducted to determine the changes in the brain imaging after FSN treatment of these 2 cases. Third, we could not provide a perfect conclusion of the possible mechanism for the FSN treatment to TN, further randomized clinical trial for mechanism discussion was required for further research.

## 4. Conclusions

FSN therapy used to be clinically effective for musculoskeletal pain, with definite curative effect and low side effects. This case report presented that FSN significantly relieved or even completely eliminated the refractory pain of TN undergone MVD. This report provides some clinical evidence regarding the efficacy of FSN on nonmusculoskeletal pain. However, the detailed mechanism of FSN on TN needs to be studied in the future.

## Acknowledgment

We are grateful to the patients for their participation in the study and allowing for publication of this case report.

## Author contributions

**Conceptualization:** Youling Gao, Jian Sun.

**Investigation:** Youling Gao.

**Methodology:** Zhonghua Fu, Li-Wei Chou.

**Project administration:** Youling Gao, Jian Sun.

**Supervision:** Jian Sun, Zhonghua Fu.

**Validation:** Youling Gao, Jian Sun.

**Visualization:** Youling Gao, Zhonghua Fu.

**Writing – original draft:** Youling Gao, Po-En Chiu.

**Writing – review & editing:** Zhonghua Fu, Li-Wei Chou.
